# A dramatic case: fatal air embolism due to intraosseous puncture

**DOI:** 10.1186/s12245-025-01066-0

**Published:** 2025-11-29

**Authors:** Maximilian Muench, Luisa Schonhart, Konrad Steinestel, Daniel Gagiannis, Josefine Christine Baudrexl

**Affiliations:** 1 Department of Pneumology, Bundeswehrkrankenhaus Ulm, Oberer Eselsberg 40, 89081 Ulm, Germany; 2 Institute of Pathology and Molecular Pathology, Bundeswehrkrankenhaus Ulm, Oberer Eselsberg 40, 89081 Ulm, Germany

**Keywords:** Emergency vascular access, Intraosseous puncture, Air embolism

## Abstract

**Background:**

Intraosseous access is a commonly used method for establishing vascular access in preclinical emergency care. It is often used when peripheral access cannot be established due to shock. Pressure infusions are used to overcome the resistance provided by the medullary cavity in order to achieve high flow rates needed for effective resuscitation. However, the literature to date does not seem to adequately describe the potential risks. In particular, air embolism is a potentially rare short-term fatal complication.

**The case:**

We report on an elderly woman who lay comatose on the floor for at least six hours before she was found and given medical attention. In the emergency room, a CT scan showed large amounts of air in her venous system. This air came from an intraosseous line in her left tibia and entered the left pelvic vein, the ventral hepatic veins, the right ventricle and the pulmonary arteries. Despite all conservative measures, the patient died 18 hours later as a result of the air embolism.

**Discussion:**

To date, there is no meaningful data in the literature on the use of pressure infusions via intraosseous access. The case described here highlights the risk of serious complications, which are probably underrepresented in the literature. There is an urgent need for self-reflection when dealing with this type of access and a necessity to develop clear guidelines regarding the inspection of connected systems and immediate emergency therapeutic measures.

**Conclusion:**

This case highlights the critical importance of careful use of intraosseous access, especially in situations with numerous other challenges that can cause distractions. Wherever possible, connection to the pressure device should be avoided rather than prescribed as an absolute requirement.

## Introduction

Intraosseous puncture and intraosseous drug and infusion therapy have developed from exceptional procedures to alternative standard procedures in recent years [1, 2]. The current guidelines of the European Resuscitation Council (ERC) and the American Heart Association (AHA) recommend intraosseous infusion as the method of choice for cardiopulmonary resuscitation in cases of delayed or failed intravenous access in both children and adults [3, 4]. Intraosseous puncture is generally a simple procedure that is safe to perform in principle. However, there is also a risk of serious complications. The guidelines on the use of intraosseous access, however, primarily deal with measures to prevent extravasation or osteomyelitis [5, 6].

## The case

**Fig. 1:**
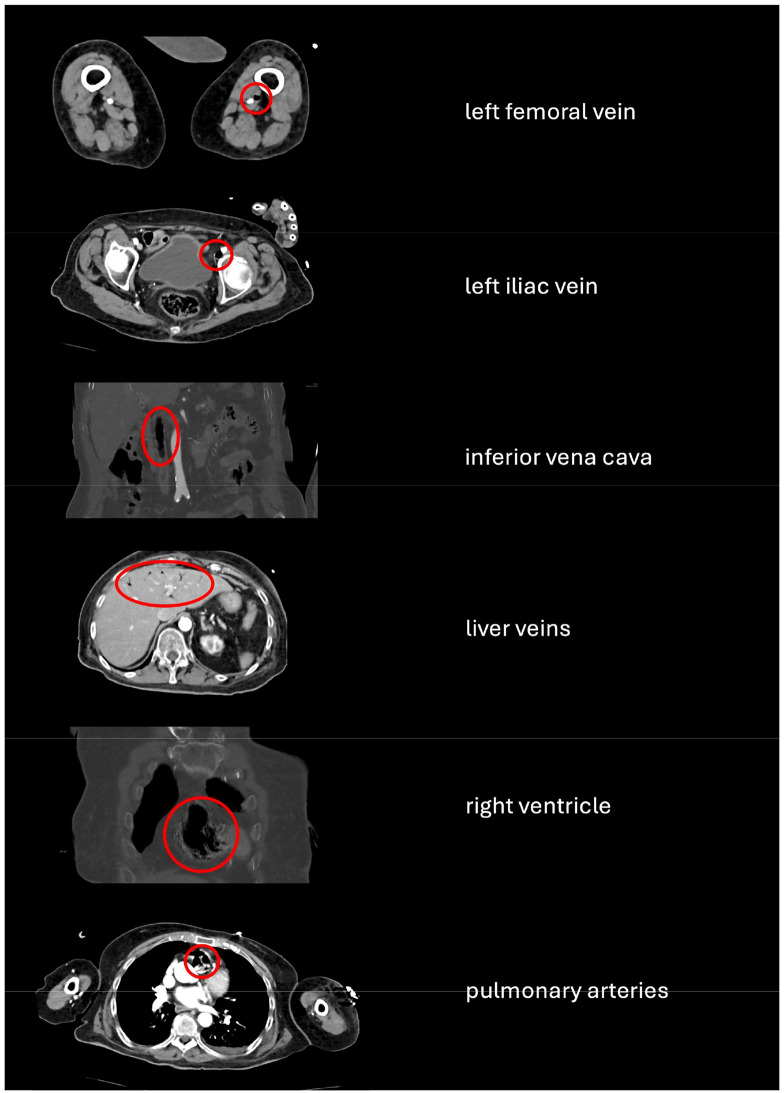
Contrast-enhanced computed tomography in the emergency room of the German armed Forces hospital in Ulm, showing air in various vessels. Axial view of the femoral, iliac and hepatic veins and the pulmonary arteries, coronary view of the right heart, sagittal view of the inferior vena cava. The red circles indicate intravascular air

**Fig. 2:**
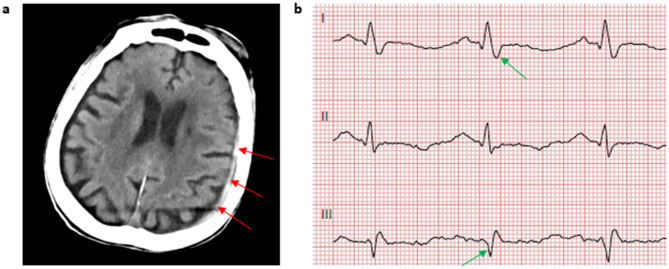
This is an overview of the most important secondary findings. In addition to a narrow subdural hematoma (**a**, red arrows), the ecg showed an S1Q3 type (**b**, green arrows)

The patient, an 88-year-old woman with a history of type 2 diabetes mellitus, severe aortic valve stenosis, heart failure, polyarthritis and normochromic normocytic anaemia, had been an irregular visitor to her doctor in the past and had shown moderate compliance with recommended treatment strategies. She was found unconscious on the bathroom floor after falling at least six hours earlier. Upon arrival of the emergency physician, based on a living will stating that mechanical resuscitation, artificial ventilation and intensive medical treatment were to be refused, oxygen was administered via a nasal tube to the patient, who was still breathing spontaneously, in order to achieve saturation levels above 90%. Due to difficult venous conditions, a 25 mm intraosseous access was promptly established in the proximal tibia on the left side, through which fluid was infused, partly as a pressure infusion, when the shock index was positive. Upon arrival at the central emergency department, the patient had a Glasgow Coma Scale score of 3. In addition to sluggish pupil reactions and spastic rales over the lungs, she had a central capillary refill time of 3 seconds, a respiratory rate of 30 breaths per minute and a heart rate of 110 beats per minute. The body temperature was 38.5 °C. The ECG showed a sinus rhythm with right bundle branch block of the S1Q3 type and preterminal T-wave inversions. The room air saturation was 91%, and blood pressure was 100/46 mmHg. The blood sugar level was significantly elevated at 623 mg/dl, lactate was 7.0 mmol/l, CRP was 58.7 mg/l, and PCT was 5.91 ng/l. Together with the documented pH value of 7.25, a potassium of 5.1 mmol/l, a sodium of 139 mmol/l, a calcium of 2.1 mmol/l, a chlorid of 94 mmol/l and an anion gap of 27.7 mmol/l, the diagnosis of ketoacidotic coma was made. The CT scan revealed a mild subdural haematoma (6 mm margin) a massive pulmonary artery air embolism extending from the pulmonary arteries via the right atrium, the inferior vena cava and the iliac veins to the left femoral vein, as well as air pockets in the ventral hepatic veins, as can be seen in Fig. 1. Figure 2 also outlines the incidental findings in the CT scan of the head and the ECG. The blood cultures quickly became positive, leading to the assumption of a septic complication. In accordance with the patient’s clear wishes, she was transferred to a normal ward for palliative care. She died 18 hours later as a result of her severely compromised state of health.

## Discussion

This case is intended to raise awareness of the risk of air embolisms caused by pressure infusions administered via intraosseous access. The method is considered safe by both experts and in the literature. However, in our opinion, the associated risks are underestimated. Of course, there are also other risk factors that can exacerbate or independently cause air embolism due to intraosseous accesses. These include head and neck surgery, especially in a sitting position, heart surgery, laparoscopic surgery, but also trauma affecting the large veins, such as the jugular vein, or the lungs. In general, injuries in an upright position promote the development of air embolism.” Given the severity of her condition (sepsis and hyperosmolar hyperglycaemic coma due to inadequately treated type 2 diabetes mellitus), the patient in our case report would certainly have died even without the complication of air embolism, especially since she had refused all intensive care treatment in advance. Nevertheless, we would like to take this course of events as an opportunity to supplement the rather minimal literature on this risk. An intensive literature search revealed only four other case reports of air embolisms following intraosseous access. Three of these cases were also fatal, although the suspected air injection was not considered the cause of death in these cases [7–9]. In another patient, the air was spontaneously reabsorbed after one day under intensive care and artificial ventilation [10]. Particularly alarming in this case is the clinical lack of correlation with the impressive amount of air. Neither the pre-hospital emergency team nor the hospital staff noticed anything unusual or deviating from standard practice, and manual pressure infusion was administered via the access as usual. In general, infusion solutions that do not contain air above the liquid level should be used. It seems obvious, but it is nevertheless worth mentioning that infusion systems must be carefully checked for complete venting, possible leaks, and potentially open three-way stopcocks before connecting them to the patient. In general, the fewer branches the system has and the shorter the overall system is, the better.” Whether the air entered through an open three-way valve, an unventilated infusion, a leaky adapter, during patient transfer or during placement cannot be determined retrospectively. Only CT imaging revealed the complicated course of events. A stronger focus on this risk in training and further investigations could potentially prevent future complications, as an air embolism alone can lead to life-threatening conditions in an otherwise uncritical patient. Causal treatment approaches are not available. In addition to preventing further air entry, the goal of treatment is to remove or at least reduce the existing air bubbles while optimizing oxygen supply. In the case of venous air embolisms, a left lateral position with the head lowered (Durant maneuver) is recommended to prevent peripheral air from entering the right heart. In the case of arterial air embolisms, a flat supine position is recommended. In both cases, the administration of 100% oxygen via a mask or intubation is recommended. This not only improves oxygen saturation, but also promotes the resorption of air bubbles (nitrogen) and reduces the volume of gas bubbles. Accompanying, generous volume administration increases venous pressure and prevents further air aspiration. For patients who are otherwise healthy and capable of being transferred, with a fulminant air embolism as outlined in the present case, pressure chamber treatment should also be discussed. This can minimize the air bubbles and achieve better tissue oxygenation. Cases such as these should be published more frequently to raise awareness of this risk.

## Conclusion

This case highlights the importance of careful use of intraosseous access. Particularly in situations where rapid action prevents a second critical assessment, it is even more important to pay attention to even minor signs of air embolism and, if necessary, to reconsider volume management. In particular, the administration of pressure infusions via intraosseous access should be avoided. In this case, it is advisable to attempt to establish a venous access again after successfully establishing intraosseous access.

## Data Availability

The data that support the findings of this study are available from the corresponding author, jb, upon reasonable request.
